# On variations in micrographic surgery and the use of horizontal histological sections in the evaluation of the surgical margin^☆^^☆☆^

**DOI:** 10.1016/j.abd.2020.02.004

**Published:** 2020-05-12

**Authors:** Anna Carolina Miola, Hélio Amante Miot, Luis Fernando Figueiredo Kopke

**Affiliations:** a Department of Dermatology, Faculdade de Medicina de Botucatu, Universidade Estadual Paulista, Botucatu, SP, Brazil; b Department of Dermatology, Hospital Universitário, Universidade Federal de Santa Catarina, Florianópolis, SC, Brazil

Dear Editor,

Complete microscopic control of the excisional margins remains the most effective method for treating non-melanoma skin tumors. Since the original idea of chemosurgery was developed by Frederic Mohs, in the 1930s, there has been substantial development of techniques for incision, inclusion, and processing of histological specimens, sectionning techniques, histological markings, and evaluation of margins. This has allowed the performance of these procedures in an outpatient setting, reducing operational time, minimizing resection of healthy tissue adjacent to the neoplasm, and reducing the cost and number of stages of surgery.^1^, ^2^

The fundamental difference between variations in micrographic surgery is the form of inspection of the involved surgical margin. Peripheral analysis techniques (*e.g*., Mohs surgery, Tübingen, the muffin technique) assess the presence of tumor cells in the hypothetical surgical border. Central analysis techniques (*e.g*., Munich), assess the entire neoplasia and its relationship with the actual surgical borders, based on the integral analysis of the excised tumor tissue sample.^3^

Portela et al. presented a technique of horizontal sectionning of the excised tissue, aiming to assess the margin compromise prior to the execution of the Mohs surgery.^4^ However, such an approach corresponds exactly to the Munich technique, described in 1995 and disseminated especially in Europe, but mentioned extensively in micrographic surgery articles, whose historical relevance cannot be disregarded.^1^, ^2^, ^3^

It should be noted that the authors make well-founded criticisms of the Mohs technique and perceive the benefits of margin control using horizontal sections, due to their experience with confocal microscopy, in addition to the emphasis on the vertical incision, which spares adjacent healthy tissue.

In fact, the modifications and advances in micrographic surgery have led to intrinsic differences in the main technical variations, which clearly favor their indications in specific situations, and whose understanding leads to the maximization of results by the micrographic surgeon.^1^, ^2^, ^3^ However, there is a lack of systematic studies (head-to-head) comparing the techniques regarding their characteristics, especially outcomes related to the surgical time, number of stages, and removal of healthy tissue. Moreover, the North American hegemony of the Mohs technique in both practice and publications has hindered dermatological science and the potential beneficiaries of the technical advances brought by the other techniques.^5^ Some particularities highlighted in the literature are listed in table 1.

**Table 1:** Comparison of the characteristics of the main variants of oncological surgery with microscopic control of the margins

	Mohs	Tübingen	Muffin	Munich
Optimal tumor size	<4 cm	>2 cm	<2 cm	<2.5 cm
Favorable excision plane	Flat or convex	Flat or convex	Flat or convex	Any
Number of histological slides^a^	Intermediate	Intermediate	Lower	Higher
Skin incision	Oblique	Vertical	Vertical	Vertical
Type of margin assessment	Peripheral	Peripheral	Peripheral	Central
Relationship of the neoplastic mass with the surgical margin	Impossible	Impossible	Impossible	Possible
Assessment of perineural invasion	More difficult	More difficult	More difficult	Easier
Resection of adjacent normal tissue	Greater^b^	Lower	Lower	Lower

a Considering an incision of the same size.

b Incision at 30°–45°.

Parallel to promoting diffusion of knowledge and research in the development of micrographic control techniques for oncological surgical margins, it is necessary to appreciate the historical merit of classically described techniques, such as the Munich technique.

## Financial support

None declared.

## Authors' contributions

Anna Carolina Miola: Approval of the final version of the manuscript; drafting and editing of the manuscript; critical review of the literature; critical review of the manuscript.

Hélio Amante Miot: Approval of the final version of the manuscript; conception and planning of the study; critical review of the literature; critical review of the manuscript.

Luis Fernando Figueiredo Kopke: Approval of the final version of the manuscript; conception and planning of the study; drafting and editing of the manuscript; critical review of the literature; critical review of the manuscript.

## Conflicts of interest

None declared.
